# Complex of HIV-1 Integrase with Cellular Ku Protein: Interaction Interface and Search for Inhibitors

**DOI:** 10.3390/ijms23062908

**Published:** 2022-03-08

**Authors:** Ekaterina Ilgova, Simon Galkin, Maria Khrenova, Marina Serebryakova, Marina Gottikh, Andrey Anisenko

**Affiliations:** 1 Chemistry Department, Lomonosov Moscow State University, 119992 Moscow, Russia; ilgova.ekaterina@gmail.com (E.I.); simon.galkin@gmail.com (S.G.); khrenova.maria@gmail.com (M.K.); gottikh@belozersky.msu.ru (M.G.); 2 Faculty of Bioengineering and Bioinformatics, Lomonosov Moscow State University, 119992 Moscow, Russia; 3 Research Centre of Biotechnology, Russian Academy of Sciences, 119071 Moscow, Russia; 4 Belozersky Institute of Physico-Chemical Biology, Lomonosov Moscow State University, 119992 Moscow, Russia; mserebr@mail.ru

**Keywords:** Ku70, HIV-1, integrase, protein–protein interactions

## Abstract

The interaction of HIV-1 integrase and the cellular Ku70 protein is necessary for HIV replication due to its positive effect on post-integration DNA repair. We have previously described in detail the Ku70 binding site within integrase. However, the integrase binding site in Ku70 remained poorly characterized. Here, using a peptide fishing assay and site-directed mutagenesis, we have identified residues I72, S73, and I76 of Ku70 as key for integrase binding. The molecular dynamics studies have revealed a possible way for IN to bind to Ku70, which is consistent with experimental data. According to this model, residues I72 and I76 of Ku70 form a “leucine zipper” with integrase residues, and, therefore, their concealment by low-molecular-weight compounds should impede the Ku70 interaction with integrase. We have identified such compounds by molecular docking and have confirmed their capacity to inhibit the formation of the integrase complex with Ku70. Our data demonstrate that the site of IN binding within Ku70 identified in the present work may be used for further search for inhibitors of the integrase binding to Ku70.

## 1. Introduction

*Human immunodeficiency virus type 1* (HIV-1) reproduction can now be controlled by specific combination antiretroviral therapy (cART). As a result, HIV-induced infection is reclassified to a manageable chronic disease [1]. However, one of the main problems for the long-term and successful usage of cART is the emergence of drug-resistant strains of the virus that require frequent drug changes [2,3,4]. Moreover, drug-resistant HIV strains can be transmitted from one patient to another, and, as a result, even newly infected patients may carry a drug-resistant virus, even though they have not yet used antiretroviral drugs [3]. To obtain drugs that do not induce the emergence of resistant strains of HIV-1 and/or possess inhibitory activity against them, various approaches are being developed, including an approach based on suppressing the interaction of viral proteins with cellular partners important for successful virus replication.

HIV-1 requires many cellular factors in order to successfully complete its replication [5,6,7], and some of them are involved in HIV-1 replication due to their direct interactions with viral proteins, e.g., HIV-1 integrase (IN) [5]. IN catalyzes the most important stage of the HIV-1 life cycle, which is the integration of the DNA copy of the viral genome into the genome of the infected cell [8]. One of the well-studied IN partners is the cellular protein LEDGF/p75, which both enhances the integration efficiency and preferentially guides HIV-1 integration to actively transcribed genome regions [9,10,11,12]. To interfere with the IN-LEDGF interaction, a family of allosteric inhibitors was recently developed and characterized, first called LEDGINs [12,13], and then ALLINIs for allosteric IN inhibitors [14], NCINIs for non-catalytic IN inhibitors [15,16], MINIs for multimerization integrase inhibitors [17], or INLAIs for integrase-LEDGF allosteric inhibitors [11]. Since the LEDGINs-binding pocket at the IN dimer interface is distant from the site of canonical inhibitor binding (e.g., raltegravir and dolutegravir), these compounds overcome resistance to classical IN inhibitors [18]. It should be noted, however, that some of these inhibitors can also inhibit post-integrational stages of viral replication [19,20,21,22]. The use of inhibitors targeting complexes of viral and cellular proteins is expected to cause a minimal chance of resistance emergence due to the high conservativeness of amino acid residues at the interaction interface [23]. This points to the promise of the approach based on suppressing the binding of IN to cellular cofactors. However, the successful development of this approach is yet limited, primarily due to our insufficient knowledge of interactions between viral and cellular proteins. Therefore, it is essential to characterize viral-cellular protein complexes that are important for viral replication and do not affect the host.

We have previously established that HIV-1 IN interacts with the human Ku70 protein, and that this interaction is extremely important for the successful repair of damages in the cell DNA resulting from the viral DNA integration [24,25]. Disruption of the IN-Ku70 interaction due to IN mutations results in a decreased replication efficiency [21]. These data give us grounds to believe that the complex of IN and Ku70 can be considered as a promising new target for developing a new generation of anti-HIV drugs. Therefore, we made an effort to determine the structure of this complex. Previously, we have found that amino acids E212 and L213 in the α6-helix of IN are important for the IN interaction with Ku70, and that their substitutions block this interaction [24]. Here, we describe our work on determining the Ku70 residues interacting with IN. Given that the functionally active IN is at least a tetramer [26], we used IN purified in the absence of detergent and in the presence of zinc ions, which was found to be mainly tetrameric [27], to evaluate IN-Ku70 binding by a pull-down assay.

Using a peptide fishing assay and site-specific mutagenesis, we have found that Ku70 residues I72, S73, I76, and, partly, S69 are crucial for IN binding, and their substitutions impede the formation of the IN/Ku70 complex. A molecular dynamics simulation was further used to elucidate the structure of the complex, and accordingly to the structure constructed, residues I72, S73, and I76 of Ku70 involved in the IN binding form one side of a pocket suitable for molecular docking. Molecular docking was applied to select small compounds that can bind at this pocket, and the compounds selected were tested using a fluorescent assay that we have previously developed for the screening of the inhibitors of IN/Ku70 binding [28]. One of the compounds tested did block Ku70 binding to IN with an IC50 of approximately 80 μM. Our data demonstrate that the selected pocket in Ku70 may be used for further search for inhibitors impeding the IN/Ku70 complex formation.

## 2. Results

### 2.1. Peptide Fishing Assay Reveals Short Peptides of the Ku70 N-Terminus That Bind to HIV-1 Integrase

**Figure 1:**
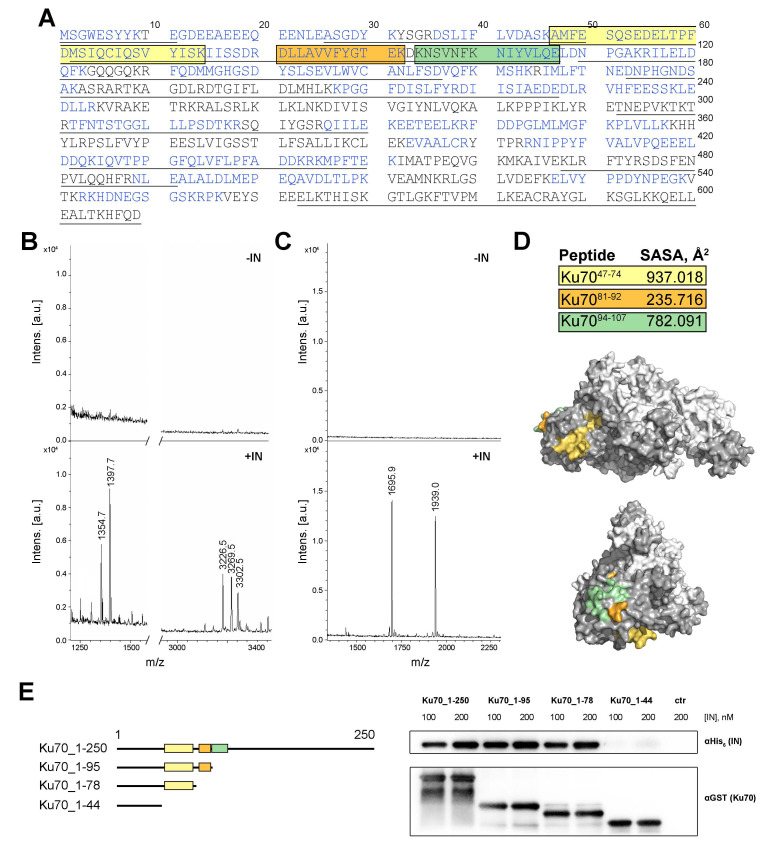
Ku70 residues 47–74 are involved in the HIV-1 integrase binding. (**A**) Ku70 amino acid sequence. Ku70 residues covered by MS-identified peptides after GluC treatment are colored in blue, and after trypsin digestion are underlined. Colored boxes indicate peptides precipitated with full-length HIV-1 IN. (**B**,**C**) MS identification of Ku70 peptides precipitated with HIV-1 IN in peptide fishing experiments. The His_6_-Ku70 was digested by trypsin (**B**) or GluC (**C**) proteases and then incubated with glutathione-agarose beads with (lower graphs, signed +IN) or without (upper graphs, signed −IN) immobilized GST-IN. The following *m*/*z* 1354.7 and 1397.7 correspond to Ku70^81–92^ and its carbamoylated form; 3226.5, 3269.5, and 3302.5 – to Ku70^47–74^, its carbamoylated form and β-mercaptoethanol adduct, respectively; 1695.9 and 1939.0 – to Ku70^94–107^ and Ku70^92–107^, respectively. (**D**) Solvent accessible surface area (SASA) values of the identified peptides, and the peptide positions in the Ku70/Ku80 heterodimer structure (PDB ID: 1JEQ). (**E**) The structure of truncated Ku70 mutants (left), and analysis of their binding to IN by GST pull-down assay (right).

The N-terminal domain of Ku70 (1–250 a.a.) is known to be sufficient for the efficient binding of HIV-1 IN [24]. However, the specific residues in this globular domain responsible for the complex formation have not been identified. For this purpose, we applied a peptide fishing assay. The full-length recombinant N-terminally His6-tagged Ku70 was subjected to digestion by trypsin or GluC protease, and, after protease inactivation, the resulting peptides were added to the GST-tagged IN immobilized on glutathione-agarose. The bound peptides were eluted and analyzed by tandem mass-spectrometry. The initial mixture of peptides was also analyzed to estimate the coverage of the N-terminal part of Ku70 by digestion products. For the trypsin digested sample, 73.23% of the full-length Ku70 and 70.4% of its N-terminal part (1–250 a.a.) were aligned with identified peptides (Figure 1A, Supplementary File S1). For the GluC digested sample, 51.56% of the full-length Ku70 and 72.4% of the N-terminal part were covered by peptides (Figure 1A, Supplementary File S2). 

In two independent experiments with trypsin- or GluC-cleaved Ku70, we identified three unique peptides bound to the immobilized IN. Peptides Ku70^47–74^ (*m*/*z* = 3226.5 and *m*/*z* = 3269.5 for its carbamoylated form and *m*/*z* = 3302.5 for β-mercaptoethanol adduct) and Ku70^81–92^ (*m*/*z* = 1354.7 and *m*/*z* = 1397.7 for its carbamoylated form) were determined after trypsin digestion, and Ku70^94–107^ (*m*/*z* = 1695.9) was observed after the GluC treatment of Ku70 (Figure 1B,C). Additionally, a peptide Ku70^92–107^ (*m*/*z* = 1939.0), which includes Ku70^94–107^, was identified (Figure 1C). We detected no significant increase in the signal-to-noise ratio in control samples without IN at the same *m*/*z* as in experiments (Figure 1B,C). All of the peptides found are located in the N-terminal domain of Ku70, which is consistent with our previous data, which show that it is this domain that ensures the Ku binding to IN [24]. 

### 2.2. Ku70^47–74^ Peptide Is Essential for IN Binding

Based on a visual inspection of the Ku70 structure (PDB ID: 1JEQ) and solvent accessible surface area (SASA) analysis, we considered that only two of the three peptides found are sufficiently solvent-exposed to contact IN (Figure 1D). The Ku70^81–92^ peptide is significantly hidden in the Ku70 structure by other residues, which may hinder its interaction with IN within the native Ku70 protein. To analyze the involvement of the detected peptides in the IN/Ku70 interaction, we prepared a set of truncated Ku70 variants, Ku70_1–250, Ku70_1–95, Ku70_1–78, and Ku70_1–3, that contain three of the peptides identified (Ku70^47–74^, Ku70^81–92^, Ku70^92–107^), two of them (Ku70^47–74^, Ku70^81–92^), one of them (Ku70^47–74^), and none of them, respectively. The position of the last residues in the deletion mutants was chosen based on the Ku70 structure (PDB ID: 1JEQ) to preserve the structure of individual elements in the investigated region of Ku70. Using a pull-down assay, we analyzed the binding of these mutants to the full-length IN, and found that Ku70_1-95 and Ku70_1–78, but not Ku70_1–33, precipitated with IN as efficiently as Ku70_1–250 (Figure 1E). Therefore, the peptides Ku70^81–92^ and Ku70^92–107^ are not required for the complex formation. In contrast, amino acids from the peptide Ku70^47–74^ are important for the interaction of IN and Ku70, since the removal of the Ku70 region from 34 to 77 a.a. disrupts the binding of Ku70 with IN. 

### 2.3. Amino Acids S69, I72, S73, and I76 of Ku70 Participate in the IN Binding

**Figure 2:**
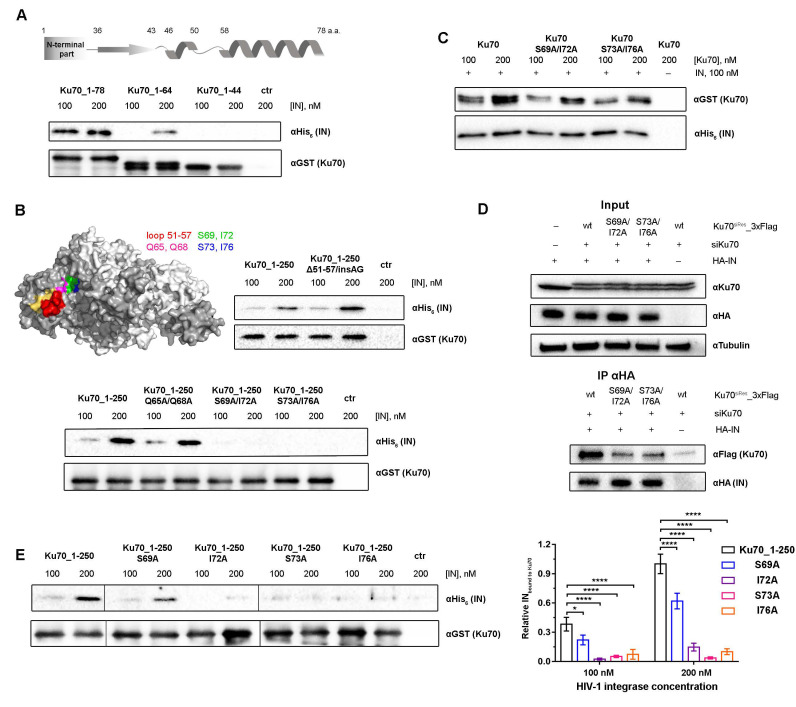
Identification of Ku70 residues involved in the HIV-1 IN binding. (**A**) Schematic representation of the Ku70_1–78 mutant secondary structure (top), and GST-pull down analysis of the interaction between truncated Ku70 mutants and HIV-1 IN (bottom). (**B**) GST-pull down analysis of the interaction between HIV-1 IN and Ku70 mutants: Ku70_1–250_Δ51–57/insAG, Ku70_1–250-Q65A/Q68A, Ku70_1–250_S69A/I72A, and Ku70_1–250_S73A/I76A. Top left insert depicts the position of Ku70 residues selected for mutagenesis in the structure of Ku70 (dark gray)/Ku80 (light gray) heterodimer (PDB ID: 1 JEQ). (**C**) His_6_ pull-down analysis of the interaction between full-length Ku70 or its double mutants and HIV-1 IN. Quantitative analysis of WB presented at Figure S1. (**D**) Immunoprecipitation of the HA-tagged IN with 3xFLAG-tagged Ku70_wt, Ku70_S69A/I72A, or Ku70_S73A/I76A in HEK293T cells. Eluates were analyzed by Western blot (bottom panel) with anti-FLAG antibodies to detect Ku70_3FLAG and anti-HA antibodies to detect HIV-1 IN. Ten percent of the cell lysates were used for input analysis by Western blot (top panel) with anti-Ku70 antibodies for the detection of Ku70 (Ku70_3xFLAG migrates slightly slower than endogenous Ku70), and anti-HA to detect HA-IN; anti-tubulin detection was used as loading control. (**E**) GST pull-down analysis of the interaction between Ku70_1–250 point mutants and HIV-1 IN by Western blot (left) and quantitative analysis (right). Mean values ± SD of three independent experiments are presented. Significance was determined by two-way ANOVA, * = adjusted *p*-value < 0.05, **** = adjusted *p*-value < 0.0001.

The peptide Ku70^47–74^ consists of an α-helix (58–74 a.a.) and a loop (50–57 a.a.) (Figure 2A) that may both participate in the formation of the IN/Ku70 interaction surface. To precisely determine which structural element is implicated in the protein–protein interaction (PPI), truncated mutants Ku70_1–64 with the defective α-helix and Ku70_1–44 without both the loop and α-helix were additionally prepared. It was shown by pull-down experiments that the destruction of the α-helix reduces the complex formation efficiency by at least four to five times, and the removal of both structural elements completely disrupts the proteins’ interaction (Figure 2A).

To check the loop involvement in the protein binding, a mutant Ku70_1–250_Δ51–57/insAG was additionally prepared, in which the loop was replaced by the AG-linker with relatively flexible Gly to preserve the protein structure. Despite the significant changes in the amino acid sequence, Ku70_1–250_Δ51–57/insAG bound to IN with the same efficiency as Ku70_1–250 (Figure 2B). Therefore, the Ku70 loop (50–57 a.a.) does not participate in PPI. 

Taking into account the fact that the removal of extended structural elements might lead to false-negative results due to some protein structural rearrangements, we further applied a point mutation approach to study the fine details of the IN/Ku70 interaction. We prepared a set of Ku70 variants containing double mutations in the α-helix: Ku70_1–250-Q65A/Q68A, Ku70_1–250_S69A/I72A, and Ku70_1–250_S73A/I76A. To check the loop involvement in the protein binding, a mutant Ku70_1–250_Δ51–57/insAG was additionally prepared, in which the loop was replaced by the AG-linker with relatively flexible Gly to preserve the protein structure. The interaction of all the mutants with IN was tested by the pull-down assay using Ku70_1–250 as a control of binding. First, we observed that, despite the significant changes in the amino acid sequence, Ku70_1–250_Δ51–57/insAG bound to IN with the same efficiency as Ku70_1–250 (Figure 2B). Therefore, the Ku70 loop (50–57 a.a.) does not participate in PPI. Second, Ku70_1–250_S69A/I72A and Ku70_1–250_S73A/I76A were unable to bind IN, whereas Ku70_1–250-Q65A/Q68A demonstrated only a 10–15% reduction in the protein binding efficiency (Figure 2B). Therefore, the residues S69, I72, S73, and I76 might be involved in PPI.

The results described above were obtained using mutants of Ku70_1–250, and, therefore, it was necessary to elucidate the effect of the tested amino acid substitutions on the binding of the full-length Ku70 and IN proteins. For this purpose, Ku70 mutants containing double mutations, Ku70_S69A/I72A and Ku70_S73A/I76A, were prepared. Both mutants were unable to efficiently bind HIV-1 IN (Figure 2C). Therefore, these residues are required for the binding of both Ku70_1–250 and full-length Ku70 to IN.

### 2.4. Double Substitutions S69A/I72A and S73A/L76A within Ku70 Impede Its Interaction with HIV-1 Integrase in 293T Cells

To reveal the biological significance of our findings, we studied whether double substitutions S69/I72 and S73/I76 influence the Ku70 capacity to bind IN in 293T cells. To reduce the level of endogenous Ku70, the cells were preliminary treated with corresponding siRNA (siKu70). For the binding analysis, we prepared plasmid vectors for the eukaryotic expression of C-terminally 3xFLAG-tagged Ku70, Ku70_S69A/I72A, and Ku70_S73/I76. In order to avoid the knockdown of these mutant Ku70 variants, synonymous substitutions were introduced in the Ku70 gene region complementary to siKu70 in the corresponding vectors, thus leading to a complementarity disruption. In addition, a plasmid vector for the eukaryotic expression of IN with HA-tag on its C-terminus was used. HEK293T cells pretreated with siKu70 were cotransfected with a combination of IN and Ku70 coding vectors, and the proteins’ binding was analyzed by immunoprecipitation, as in [24]. FLAG-tagged Ku70 was found to readily co-precipitate with IN, whereas the binding of Ku70_S69A/I72A and Ku70_S73A/I76A with IN was significantly impaired (Figure 2D). We could therefore conclude that Ku70 amino acids located in the α-helix (58–74 a.a.) are necessary for IN binding, both in vitro and in vivo.

### 2.5. Elucidation of Ku70 Amino Acid Residues Involved in Integrase Binding

To verify the significance of each amino acid residue within the Ku70 α-helix for IN binding, we additionally constructed Ku70_1–250 variants with single mutations: Ku70_1–250_S69A, Ku70_1–250_I72A, Ku70_1–250_S73A, and Ku70_1–250_I76A. All point mutants were tested for their binding with HIV-1 IN, and it was found that the Ku70_1–250_S69A mutant retained its IN binding activity at the level of 55-73% of the wild-type protein, whereas Ku70_1–250_I72A, Ku70_1–250_S73A, and Ku70_1–250_I76A mutants did not form a detectable complex with IN (Figure 2E). This result demonstrates that amino acids I72, S73, and I76 probably form the IN binding site in the Ku70 structure, whereas S69 may additionally stabilize the Ku70/IN complex, but is not crucial for PPI.

### 2.6. Investigation of the IN/Ku70 Complex Structure Using Molecular Dynamic Approach

To specify the binding site in the IN/Ku70 complex with the atomic resolution, we applied classical molecular dynamic (MD) simulations. We started with the preparation of the full-atom model of the soluble form of the IN dimer from the available X-ray structure PDB ID: 1EX4 [29]. Recent cryo-EM studies demonstrate different orientations of the C-terminal domains (CTDs) relative to the catalytic core domain (CCD) and α6 helix [30]. Therefore, we performed a 250 ns MD simulation of the IN dimer in a rectangular water box. We found that the angle between α6 helixes remains large and that CTDs do not tend to dimerize similarly to the X-ray structure PDB ID: 1EX4. Residues 221–224 from IN are rather flexible according to the MD simulations, and CTDs-relative orientations in cryo-EM experiments might be due to the specific experimental conditions that are not reproduced in solution experiments in silico. Therefore, we mostly rely on the CTDs locations in the IN dimer, as in the PDB ID: 1EX4 [29]. It is known that retroviral integration is realized by intasomes comprising an integrase tetramer, octamer, or even dodecamer tightly associated with viral DNA ends [26,31]. However, those are organized in such a way that the region between α6 helixes of two monomers remains free even at higher oligomerization states. Therefore, the dimeric state of HIV-1 IN is sufficient for modeling of the 3D structure of the IN complex with the Ku heterodimer, based on the experimental data on the crucial role of E212 and L213 in the complex formation [24].

**Figure 3:**
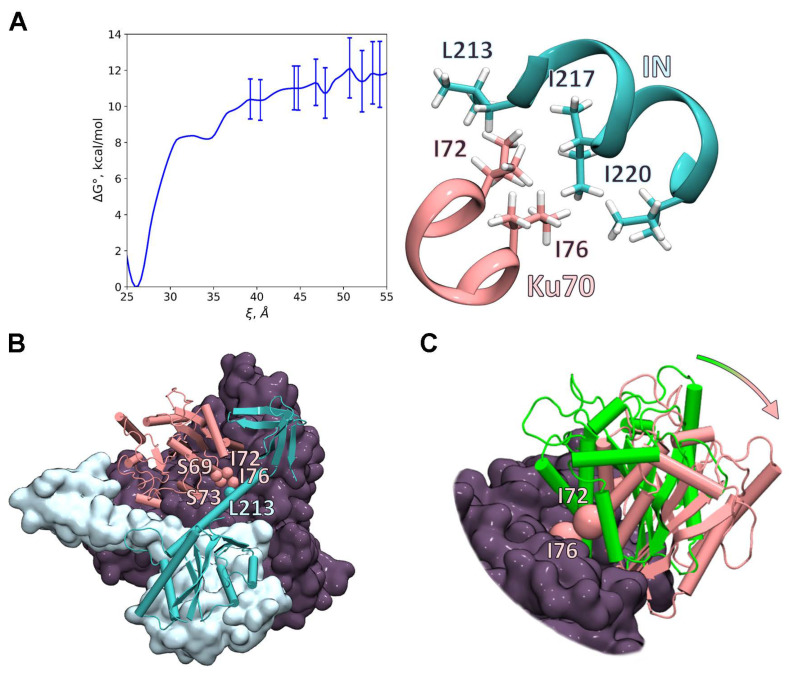
Three-dimensional model of the Ku70/IN complex. (**A**) Gibbs energy profile of the N-terminal domain of Ku70 and IN binding. The error bars are calculated using umbrella integration approach. The inset demonstrates the hydrophobic binding motif of the so-called “leucine zipper”. Ku70 and IN carbon atoms are colored pink and cyan, respectively, and hydrogen atoms are white. (**B**) Calculated structure of the Ku70/Ku80 heterodimer and IN dimer complex. IN dimer is shown in light blue: the monomer that interacts with the N-terminal domain (up to 250th residue) of Ku70 is shown in cartoon representation. The dark isosurface is the Ku70/Ku80 heterodimer, except the N-terminal domain of the Ku70, which is shown in pink cartoon representation. (**C**) Alignment of the crystal structure of Ku70/Ku80 heterodimer (PDB ID: 1JEQ [33]) and the calculated structure. The N-terminal domain of Ku70 from the PDB ID: 1JEQ is shown in green. The arrow shows the rotation direction of the N-terminal Ku70 domain.

We followed the consecution of experiments and started with the complex of IN with the truncated Ku70_34–250 protein. We prepared a set of initial structures with different initial orientation of the proteins. When choosing initial sets of coordinates, we relied on experimental data on critical mutations in Ku70 reported in this study and IN mutations reported in [24]. Accordingly, important residues from Ku70 are I72, S73, and I76 (Figure 2E). For IN, even a single mutation of E212 or L213 has a severe destructive effect on the complex formation [24]; therefore, these residues are supposed to be involved in the complex formation. All initial structures were obtained in such a manner that the selected residues of the Ku70 and IN are separated by a water layer that is one to two water molecules thick to avoid direct contact between residues. The latter was carried out to be sure that the Ku70_34–250–IN interface is simultaneously formed during the MD simulation if two proteins are located close to each other. All initial complexes had the same binding motif after MD simulations (Figure 3A). It is similar to a so-called “leucine zipper” [32] and is composed of I72 and I76 of Ku70 and L213, I217, and I220 of IN. The L213 of IN mostly interacts with the Ku70 residues, which is in line with its experimentally observed importance for the complex formation [24]. The role of the S73 is not that evident. The structural analysis demonstrates that the S73 side chain forms a hydrogen bond with the CO group of the S69 main chain, which stabilizes the α-helix of Ku70. In addition, it forms a stable hydrogen bond with the positively charged side chain of the R247 of Ku70, which also stabilizes the structure. 

To quantify these interactions, we calculated the Gibbs energy profile of the complex formation (Figure 3A). We chose a collective variable ξ as a distance between the carbon atom of the backbone of Ku70 Phe40 and a carbon atom of the backbone of IN E212. We observed the minimum at ξ = 26 Å that corresponds to the bound state. The Gibbs energy gradually increased with the increase in the collective variable. The plateau corresponding to the dissociated complex began at ξ = 40 Å with a relative energy of 10.3 kcal/mol. We intentionally continued simulations and increased the reaction coordinate up to 55 Å to be sure that we observed the complete dissociation. We observed a set of minima and maxima at the reaction coordinate values between 40 and 55 Å. The energies of minima in this region differ less than 1.5 kcal/mol, which is within the error range calculated with the umbrella integration approach. Therefore, we might take an approximate mean value of 11 kcal/mol of dissociation Gibbs energy and estimate the corresponding dissociation constant at 300 K, Kd = 10 nM. This is in good agreement with the experimentally observed 100 nM [24], which further supports the binding mechanism proposed in this study.

In vivo Ku70 exists as a heterodimer with Ku80. Therefore, in vivo IN is likely to form a complex with the Ku70/Ku80 heterodimer rather than with Ku70. This means that the binding site found for the truncated Ku70 should also be available for binding with the Ku70/Ku80. We constructed the complex of the Ku70/Ku80 heterodimer and IN dimer using motifs of the truncated complex discussed above (Figure 3). To carry this out, we rotated the N-terminal domain of Ku70 outward (Figure 3C). The same conformational flexibility is already observed experimentally for the structurally similar vWA domain of Ku80 upon X-KBM peptide binding [34]. After 50 ns equilibration and a 200 ns production MD run, the binding site remained the same as for the truncated complex (Supplementary File S3, Figure 3C). To conclude, the main contribution for the Ku70/IN complex formation is the “leucine zipper” formed by I72 and I76 from Ku70 and L213, I217, and I220 from IN.

### 2.7. Low-Molecular-Weight Compounds Shielding Residues S69, I72, S73, and I76 in Ku70 Disrupt Its Interaction with IN

**Figure 4:**
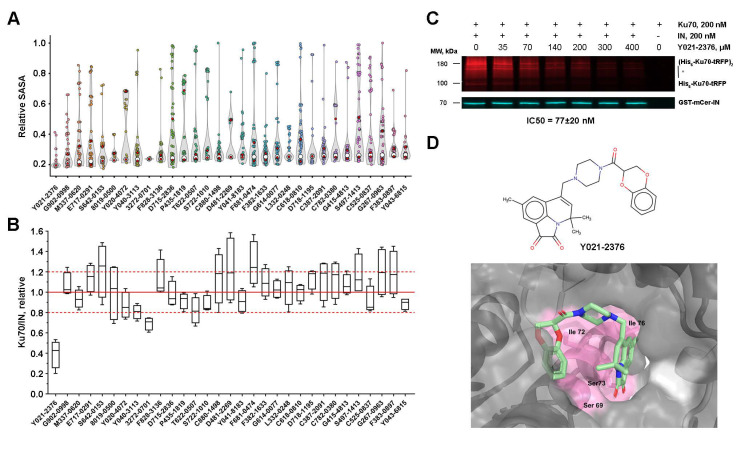
Y021-2376 blocks HIV-1 IN binding to Ku70. (**A**) Distribution of relative SASA S69 + I72 + S73 + I76 calculated for complexes of the Ku heterodimer with top 31 investigated compounds. White dots correspond to the median of the distributions. (**B**) The effect of 100 μM compounds on the Ku70/IN complex formation. (**C**) Fluorescent pull-down assay analysis of the interaction of His6-Ku70-tRFP (200 nM) and GST-mCer-IN (200 nM) in the presence of an increasing concentration of Y021-2376. (**D**) Chemical structure of the most active compound Y021-2376 (top panel) and probable inhibitor position in the Ku70/Ku80 heterodimer (bottom panel). Ku70 residues involved in HIV-1 IN binding are pink. Ku70 surface is colored in dark gray and Ku80 in light gray.

The Ku70 residues S69, I72, S73, and I76, important for the IN binding, are part of a pocket in the Ku heterodimer structure, suitable for molecular docking (Figure S2A). For docking studies, we chose the Ku heterodimer instead of Ku70 since it is the heterodimer that is involved in post-integrational DNA repair [25]. However, given that the heterodimer is recruited to the repair site due to the binding of IN to Ku70, we considered that small compounds targeting a pocket in the heterodimer should also be active against the Ku70/IN complex. To select such compounds, the representative Diversity Library containing 50,000 compounds with high diversity scoring (ChemDiv, Moscow, Russia) was used for docking into the Ku heterodimer pocket (PDB ID: 1JEQ). The ligands and receptor were prepared for AutoDock-GPU and 25 runs were performed for each compound. All compounds were ranked based on the calculated minimal binding free energy, and only the first 444 compounds with a minimal free energy less than −11 kcal/mol were selected for an additional round of docking with 100 runs for each compound. The compounds were ranked based on their ability to hide the target residues. For this purpose, SASA values of S69, I72, S73, and I76 residues were calculated for the Ku complexes with each compound. Based on this scoring, 31 compounds with the highest rank were selected for further testing in vitro (Figure 4A and Table S1). All of the selected compounds efficiently hid the selected residues: their median SASA values less than 24.62 Å^2^ were observed for the Ku/compound complexes compared to 126.57 Å^2^ for the same residues in the structure of Ku alone.

To evaluate the inhibition properties of the selected compounds, the fluorescent pull-down assay described previously was used [28]. First, all of the compounds were tested at a 100 μM concentration in triplicates. Only 2 of 31 compounds decreased the relative amount of Ku70 bound to IN to 80% or less (Figure 4B). Second, the most active compound, named Y021-2376, was titrated to estimate IC50. It was found to be 77 ± 20 μM (Figure 3C). After an exhaustive-ranking docking, this compound clustered in three main positions (Figure S2). The most represented position (147/150 runs) is shown in Figure 4D. Considering the too-high IC50 value of this compound, we did not test its inhibition properties on the cellular single-round replication model of HIV-1 in cell culture. However, our data demonstrate that the site of IN binding within Ku70 identified in this work may be used for a further search for inhibitors of the IN/Ku70 binding. 

## 3. Discussion

With the approval of raltegravir for the treatment of AIDS, HIV-1 integrase has joined the group of viral proteins targeted by anti-HIV drugs [35]. Unfortunately, raltegravir has a relatively low genetic barrier to resistance [36]. Integrase inhibitor dolutegravir is effective against many drug-resistant HIV-1 strains [37], and a new inhibitor cabotegravir has a prolonged effect: its injections, in combination with a non-nucleoside reverse transcriptase inhibitor rilpivirine every 4 or 8 weeks, allow for the maintenance of a viral load at an undetectable level in 90% of HIV-1-infected patients [38]. Nonetheless, over time, drug resistance develops, even to such efficient IN inhibitors [36,39]. Thus, there is a clear need for the development of new anti-HIV drugs, especially those not or minimally causing the emergence of resistant strains. 

These can be compounds targeting cellular proteins involved in HIV-1 replication, or their complexes with viral proteins. Cellular proteins are not under the genetic control of the viral genome, and, hence, compounds targeting host proteins possess a much higher genetic barrier to drug resistance compared with drugs that act on viral proteins [23,40]. This is the reason for the search for IN cellular partners and the development of integration inhibitors that target the complexes of these partners with IN [41,42].

We have previously demonstrated that the HIV-1 IN interaction with the cellular Ku70 protein is important for efficient viral replication, since IN through direct binding with Ku70 recruits a whole DNA-PK complex to the viral DNA integration site. DNA-PK then activates downstream mediators and initiates the repair of the post-integrational gaps in pro-viral DNA. An efficient DNA repair enables HIV-1 to pass through the viral life cycle [25]. The key point in this model of post-integration repair is the binding of IN to Ku70, and, therefore, the disruption of this binding by small molecules makes it possible to block HIV-1 replication. However, in order to develop molecules capable of disrupting this binding, we need to know the structure of the protein–protein interaction interface. It has been shown that IN residues E212 and L213 are involved in the Ku70 binding, and that the N-terminal domain of Ku70 interacts with IN [24]. 

Here, we continued the study of the IN interaction with Ku70 and determined Ku70 residues crucial for the IN binding. Based on the results of the peptide fishing experiment, site-directed mutagenesis, and pull-down assays, we could suppose that amino acids I72, S73, and I76 probably form the IN binding site in the Ku70 structure, whereas S69 may additionally stabilize the Ku70/IN complex, but is not essential for the proteins’ interaction. The substitution of these amino acids had a strong negative effect on the Ku70 capacity to bind IN, both in the case of recombinant proteins and proteins expressed in 293T cells. 

To clarify the structure of the binding site in the IN/Ku70 complex, a classical molecular dynamic simulations approach was further applied. As a result, we could conclude that the main contribution for the Ku70/IN complex formation is made by the “leucine zipper” formed by I72 and I76 from Ku70 and L213, I217, and I220 from IN. This binding motif is supported by the calculated dissociation constant, which is in agreement with the experimental value. The hydrophobic binding leucine-rich interface is widely spread in the heterodimers. For example, the heterodimer interface in the GABA_B_ receptor is constructed similarly to the Ku70/IN interface; couples of leucine residues from the α-helices of both monomers form hydrophobic contacts [43]. The binding power of the leucine zipper systems is shown on the artificial heterodimerizing leucine zipper systems, which demonstrate dissociation constants up to 10^−15^ M [44]. Therefore, even a compact binding interface composed of five hydrophobic amino acid residues can be responsible for the complex formation.

The elucidation of the amino acid residues involved in the IN/Ku70 complex formation allowed us to search for compounds capable of destroying the complex. Using molecular docking, we could search for compounds targeting both IN and Ku70. We considered that it would be unreasonable to take IN as a target because all residues involved in the Ku70 binding are located in a long α-helix, in the structure of which, there is no pocket suitable for docking. Earlier, we showed that the conjugate of an 11-mer oligonucleotide with eosin can bind to IN and the oligonucleotide part shields E212 and L213 residues, thus blocking the IN binding to Ku70 [24]. However, the conjugate binding to IN occurred mainly due to the eosin residue, which binds in a pocket located in the C-terminal domain of IN. In the case of small molecules, such a strong displacement of their binding site from the residues E212, L213, I217, and I220 is impossible. Therefore, we carried out molecular docking into the pocket within the Ku heterodimer formed by the residues S69, I72, S73, and I76 of Ku70. Thirty-one compounds with the best characteristics were selected, and their capacity to inhibit the interaction of IN and Ku70 was tested using the previously described fluorescent pull-down assay [28]. The most active compound Y021-2376 had an IC_50_ of approximately 80 μM. 

Obviously, the inhibitory activity of this compound is too low to be tested as an inhibitor of HIV-1 replication in cell culture. Nevertheless, we believe that the site of IN binding within Ku70 identified in this work may be used for a further search for inhibitors of the IN/Ku70 interaction, and we have at least two reasons for this conclusion. First, the pocket selected for the docking of potential inhibitors is formed mainly by the Ku70 residues; however, the Ku80 region can also be involved in the formation of the pocket (Figure 4D). We realize that, by using the Ku heterodimer for docking instead of the Ku70 subunit, we might have missed some active inhibitors of the Ku70/IN complex, but we believe that, due to this approach, the inhibitors found have greater biological relevance and a higher potential for further optimization and in vivo application. The second and most important reason deals with the position of the pocket: it is located far from the regions of Ku70 participating in its binding to both other DNA-PK subunits, Ku80, and DNA-PKcs, and to DNA. Therefore, we can consider that the binding of inhibitors of the Ku70 interaction with HIV-1 IN will not interrupt Ku70 functions essential for cell survival. 

## 4. Materials and Methods

### 4.1. Oligonucleotides and Plasmids

Plasmid constructs pGEX-6p1-Ku70_1–78, pGEX-6p1-Ku70_1–64, and pGEX-6p1-Ku70_1–44 were generated from pGEX-6p-1-Ku70_1–250 construct by the addition of a STOP-codon by site-directed mutagenesis with primers Ku70–78_STOP/Ku70–78_STOP_anti, Ku70–64_STOP/Ku70–64_STOP_anti, and Ku70–44_STOP/Ku70–44_STOP_anti, respectively (Table S1). Construct pGEX-6p1-Ku70_1–250_Δ51–57/insAG was produced from pGEX-6p1-Ku70_1–250 by the addition of AlaGly-coding-linker instead of 51–57 a.a. using primer pair Ku70_Δ51–57/insAG/Ku70_Δ51–57/insAG_anti by site-directed mutagenesis. Plasmids coding for double or single Ku70 mutants pGEX-6p1-Ku70_1–250_Q65A/Q68A, pGEX-6p1-Ku70_1–250_S69A/I72A, pGEX-6p1-Ku70_1–250_S73A/I76A, pGEX-6p1-Ku70_1–250_S69A, pGEX-6p1-Ku70_1–250_I72A, pGEX-6p1-Ku70_1–250_S73A, and pGEX-6p1-Ku70_1–250_I76A were prepared from the construct pGEX-6p1-Ku70_1–250 by site-directed mutagenesis with primer pairs Ku70_Q65A/Q68A/Ku70_Q65A/Q68A_anti, Ku70_S69A/I72A/Ku70_S69A/I72A_anti, Ku70_S73A/I76A/Ku70_S73A/I76A_anti, Ku70_S69A/Ku70_S69A_anti, Ku70_I72A/Ku70_1–250_I72A_anti, Ku70_S73A/Ku70_S73A_anti, and Ku70_I76A/Ku70_I76A_anti, respectively. Plasmids coding for double Ku70 mutants pGEX-6p1-Ku70_S69A/I72A, and pGEX-6p1-Ku70_S73A/I76A were prepared from the construct pGEX-6p1-Ku70 by site-directed mutagenesis with primer pairs Ku70_S69A/I72A/Ku70_S69A/I72A_anti and Ku70_S73A/I76A/Ku70_S73A/I76A_anti. Site directed mutagenesis was performed using Quick Change II Site-Directed Mutagenesis Kit, Agilent Technologies, Santa Clara, CA, USA.

### 4.2. Recombinant Proteins Expression and Purification

HIV-1 integrase carrying N-terminal His_6_-tag was expressed and purified as previously described [45]. All Ku70 proteins carrying N-terminal GST-tag were purified as previously described [46]. GST-mCer-IN and His_6_-Ku70-tRFP were expressed and purified in the same way as His_6_-IN and GST-Ku70, respectively, as previously described in [45,46].

### 4.3. Peptide Fishing Assay

One milligram of His_6_-Ku70 immobilized on Ni-NTA-agarose was digested overnight at 37 °C by trypsin (40:1 *w*/*w*, Promega, Madison, WI, USA) or at 55 °C by GluC protease from *Bacillus intermedius*, strain 3-19 (40:1 *w*/*w*, a kind gift from Dr. G. Rudenskaya) [47] in 20 mM Hepes pH 7.5, 100 mM NaCl, 7.5 mM MgCl2, 2 mM 2-merkaptoethanol, and 0.1% NP40. The proteases were inactivated by addition of Halt™ Protease Inhibitor Cocktail (Thermo Fisher Scientific, Waltham, MA, USA). The mixtures obtained (final concentration 1 µM equivalent of initial Ku70) were incubated for 1 h at 25 °C with His_6_-IN (500 nM) in 500 µL of buffer A (20 mM Hepes pH 7.5, 100 mM NaCl, 7.5 mM MgCl2, 2 mM 2-merkaptoethanol, 50 μg/mL BSA, and 0.1% NP40) with 30 mM imidazole. Then, 30 μL Ni-NTA-agarose beads were added to the reaction mixtures, followed by 1 h incubation at room temperature under rotation. Beads were washed twice with washing buffer (buffer A without BSA). The peptides were eluted by 7 M urea at 94 °C for 5 min. Ten microliters of the sample were desalted on a Millipore ziptip C18 P10 cartridge. The peptides for mass spectrometric analysis were eluted with 50% acetonitrile 0.1% trifluoroacetic acid (TFA) solution. Matrix-assisted laser desorption ionization time-of-flight MS analysis of total digests and bound peptides was performed on an UltrafleXtreme MALDI-TOF/TOF mass spectrometer (Bruker Daltonics, Bremen, Germany) by detection of MH+ molecular ions. An aliquot of 0.5 μL of sample was mixed with 1 μL of 2,5-dihydroxybenzoic acid solution (40 mg/mL in 30% acetonitrile, 0.5% TFA). The spectra were recorded in reflector mode, and the accuracy of the monoisotopic mass peak measurement was within 50 ppm. Mass spectra were processed using FlexAnalysis 3.3 software (Bruker Daltonics, Bremen, Germany). Proteins were identified using the home database, which was preloaded with sequences of proteins under study and Mascot combined peptide mass fingerprint + MS/MS search program (Mascot version 2.3.02).

### 4.4. Protein Binding Assays

To detect interactions between IN and Ku70 or their mutants, the GST pull-down and His6 pull-down assays were performed. IN and Ku70 were incubated in 150 μL of buffer A (20 mM Hepes pH 7.5, 100 mM NaCl, 7.5 mM MgCl2, 2 mM 2-merkaptoethanol, 50 μg/mL BSA, and 0.1% NP40; in case of His6 pull-down, 30 mM imidazole was added) at room temperature for 1 h. The 100 nM GST-Ku70 and 100 or 200 nM His_6_-IN were used for GST pull-down, and 100 nM His_6_-IN and 100 or 200 nM GST-Ku70 were used for His_6_ pull-down. We used such protein concentrations to precisely detect a difference in their ability to form the protein–protein complex, due to Kd for IN-Ku70 being determined as approximately 100 nM. At higher concentrations, there may be no difference due to saturation. Then, 20 μL of glutathione-agarose (for GST pull-down) or Ni-NTA-agarose beads (for His6 pull-down) was added to the reaction mixtures, followed by 1 h incubation at room temperature under rotation. Beads were washed twice with washing buffer (buffer A without BSA). The proteins were eluted from the beads with 20 μL of 1X SDS-PAGE loading buffer at 95 °C for 5 min and analyzed by SDS-PAGE with subsequent Western blotting. In parallel, a non-specific binding of the prey protein was analyzed by the addition of GST-tagged Ku70 samples to Ni-NTA-agarose or His6-tagged IN proteins to glutathione-agarose without the presence of respective bait. The pull-down of an individual GST protein was used as a control for a non-specific IN binding to GST.

### 4.5. Co-Immunoprecipitation

Then, 1 × 10^6^ 293T cells were transfected with 50 nM siKu70 or siCntr (Table S2) using RNAiMAX (Invitrogen, Waltham, MA, USA), and, 24 h later, by 9 μg of empty pCDNA3.1 vector, or were cotransfected with 3 μg of pCDNA3_Ku70_3xFLAG_siRes, pCDNA3_Ku70_3xFLAG_S69A/I72A_siRes, or pCDNA3_Ku70_3xFLAG_S73A/I76A_siRes and 6 μg of pCDNA3_IN_HA or empty pCDNA3.1 vectors using the Lipofectamine 3000 (Invitrogen). Forty-eight hours after, second transfection cells were lysed for 30 min on ice in RPMI medium (Invitrogen) supplemented with protease inhibitor cocktail (Thermo Fisher Scientific, Waltham, MA, USA) and 0.25% NP-40 (Helicon). Lysates were cleared by centrifugation for 10 min at 14,000 rpm and protein concentration was measured using DC Protein Assay (Bio-Rad, Hercules, CA, USA). A total of 0.1 mg of cell lysates was saved for input analysis. One milligram of total protein was mixed with HA-antibody conjugated agarose (Sigma, Saint Louis, MO, USA) and incubated for 5 h at 4 °C. The beads were washed 4 times with lysis buffer and bound proteins were eluted with HA-peptide for 25 min at 37 °C. Elution fractions and inputs were then analyzed by Western blot (see above).

### 4.6. Western Blot Analysis

Protein samples were separated by 12% SDS PAGE and analyzed for the presence of GST- or His_6_-tag by WB with rabbit anti-GST (Sigma) and mouse anti-His6 antibodies (Sigma), respectively. For the detection of IN_HA, an anti-HA monoclonal antibody (Invitrogen) was used. For the detection of Ku70_3FLAG, an anti-FLAG M2 HRP-conjugated antibody (Sigma) or anti-Ku70 rabbit antibody (Sigma) were used. Mouse anti-human tubulin clone 12G10 mAb (Developmental Studies Hybridoma Bank at the University of Iowa) as primary antibodies was used. HRP-conjugated anti-rabbit (Sigma) and anti-mouse antibodies (Sigma) were used as secondary antibodies. Visualization of specific protein bands was performed with Clarity Western ECL substrate (Bio-Rad) on ChemiDoc MP system (Bio-Rad).

### 4.7. Fluorescence Gel Imaging

To investigate the influence of inhibitors on the His_6_-Ku70-tRFP/GST-mCer-IN complex stability, compounds were incubated in concentrations of 100 μM and in increasing concentration in case of the most active compound, Y021-2376, with 200nM GST-mCer-IN and 200nM His_6_-Ku70-tRFP in 150 μL of buffer A at room temperature for 1 h. Then, the complexes His_6_-Ku70-tRFP/GST-mCer-IN and free GST-mCer-IN were precipitated by glutathione-agarose as described above. After elution of proteins with 20 μL of 1X SDS-PAGE, the levels of His6-Ku70-tRFP and GST-mCer-IN were analyzed by standard SDS-PAGE electrophoresis, with subsequent detection of fluorescence in the gel (see Fluorescence Imaging subsection). The fluorescence signals ratios (tRFP/mCer) were used as a measure of protein-binding efficiency. This ratio in the absence of the inhibitor was taken as 100%.

### 4.8. Fluorescence Imaging

Fluorescent signals were measured in gel using the ChemiDoc MP system (Bio-Rad). In the case of gel fluorescence measuring, 530/28 and 605/50 nm emission filters and Blue Epi illumination and Green Epi illumination excitation sources were used for mCer and tRFP, respectively. 

### 4.9. Molecular Dynamic Simulations

All unconstrained classical molecular dynamic (MD) simulations were performed with the same protocol using NAMD software [48]. Protein or protein complexes were solvated in the rectangular water box so that the distance from the protein to the cell border was not less than 12 Å. The systems were neutralized by adding sodium or chloride ions. First, 10,000-step energy minimizations were performed. All MD simulations were performed in the NPT ensemble at T = 300 K and p = 1 atm. Langevin dynamics were used for the temperature control. Constant pressure was achieved using a modified Nosé–Hoover method, in which, Langevin dynamics were used to control fluctuations in the barostat [49,50]. Preliminary equilibration of model systems was performed for 50 ns. The production run lengths were 200 ns. The CHARMM36 [51] force filed parameters were utilized for protein molecules, and TIP3P [52] for water. Analysis of all 3D structures was performed in VMD software package [53].

The PDB ID: 1EX4 crystal structure of HIV-1, IN, was utilized as a template for MD simulations of its wild type (WT) and mutant forms [29]. The available X-ray data were referred to the IN with increased solubility due to the point mutations C56S, W131D, F139D, F185K, and C280S. We substituted these residues back to the WT ones in our model system and reconstructed amino acid residues that were not resolved in the crystal structure. The 250 ns MD simulation was performed to obtain an equilibrated structure of the IN dimer. 

The model system mimicking interactions of the truncated Ku70 with the integrase was constructed as follows. We extracted the N-terminal part up to 250th residue of the Ku70 from the PDB ID: 1JEQ structure [33]. The wild type IN was oriented relative to Ku70 so that the residues that were found to be important for complex formation were close to each other. These residues for Ku70 are reported in this study and for IN in ref [33]. We performed a set of 8 runs, starting with different relative orientations of Ku70 and IN. Some of these complexes dissociated, but others formed stable interactions and were the same. This complex was utilized as initially for the estimates of binding energy. The reaction coordinate was set as a distance between the carbon atom of the backbone of Ku70 Phe40 and a carbon atom of the backbone of IN Glu212. A set of MD runs was performed with the ½K·(ξ − ξ_0_)^2^ harmonic potentials centered at different reaction coordinate values, ξ_0_ = 26 to 54 Å, with 2 Å increment, with the force constant K = 2 kcal/(mol·Å^2^). Trajectories were simulated sequentially, starting with the smallest value. The last frame of the run with the ξ_0,i_ was utilized as the initial structure for the constrained run, with the reaction coordinate centered at the next ξ_0,i_ + 2 Å value. This was carried out to serve better relaxation of the system. Each MD trajectory was 20 ns in length. The data analysis was performed using umbrella integration (UI) and weighted histogram analysis methods (WHAM). The reaction coordinate was divided into 200 bins for statistical analysis, and, first, 10 ns. 

The model of the complex between IN and truncated Ku70 was utilized to construct the full-length complex of an IN dimer and a heterodimer of Ku70/Ku80. The Ku70/Ku80 heterodimer was obtained from PDB ID: 1JEQ crystal structure [33]. The N-terminal Ku70 domain was outward rotated to eliminate steric hindrance caused by the IN binding. This is in line with the experimentally observed outward rotation of the vWA domain of Ku80 caused by X-KBM peptide binding [34]. The IN dimer was added to the model system according to the structural data obtained on the previous step, when calculating Gibbs energy profile of complex formation. 

### 4.10. Molecular Docking 

#### 4.10.1. System Configuration and Computational Details

This research was performed using an AutoDock-GPU (v1.4.3), OpenCL and Cuda accelerated version of AutoDock4.2.6 [54] installed in macOS Catalina 10.15.7 with 16 GB RAM and AMD Radeon Pro 5300M 4 GB graphics card. The 3D visualization images were created using PyMol. Data analysis was performed using Python.

#### 4.10.2. Protein Model Preparation

The X-ray crystal structure of human Ku heterodimer (PDB ID: 1JEQ) was downloaded from Protein Data Bank [33]. All water molecules were removed, and AutoDockTools software [55] was used to prepare the required files for AutoDock-GPU by assigning hydrogen polaraties, calculating Gasteiger charges to protein structures, and converting protein structures from the PDB file format to PDBQT format. Energy grid maps were calculated using AutoGrid program [55]. A grid size was set to 82 × 72 × 76 (x, y and z) points with a spacing of 0.292 Å. The grid center was designated at x, y, and z dimensions of 41.744, 8.608, and 128.236, respectively.

#### 4.10.3. Ligand Preparation

A total of 50,000 molecules from ChemDiv Diversity Library were selected for structure-based virtual screening against HIV-1 integrase putative binding site of Ku heterodimer. Virtual compound library in SMILES format was converted to PDB format using an open-source program for preparing small-molecule libraries Gypsum-DL [56]. All of the hydrogen atoms were added to the ligand molecules. Ligand ionization states were generated at pH 7.2.

#### 4.10.4. First Docking Iteration

First docking iteration calculations of 50,000 molecules library were carried out with the following parameters: 25 LGA runs, 2,500,000 score evaluations (max.) per LGA run, 27,000 generations (max.) per LGA run, ADADELTA local-search method, 300 local-search iterations (max.), 150 population size, 2% mutation rate, 80% crossover rate, 80% local-search rate, and 60% tournament (selection) rate.

#### 4.10.5. Second Docking Iteration

After the first docking iteration compounds were sorted by their calculated best minimal binding free energy, −11 kcal/mol cutoff was chosen. As a result, 444 compounds were subjected to the second docking iteration with the following parameters: 100 LGA runs, 2,500,000 score evaluations (max.) per LGA run, 42,000 generations (max.) per LGA run, ADADELTA local-search method, 300 local-search iterations (max.), 150 population size, 2% mutation rate, 80% crossover rate, 80% local-search rate, and 60% tournament (selection) rate.

#### 4.10.6. SASA Calculation

SASAs of Ku70 residues S69, I72, S73, and I76, with or without docked compounds, were calculated using FreeSASA software [57]. 

#### 4.10.7. In Silico PAINS Filtering

All compounds in ChemDiv diversity dataset were filtered for PAINS [58]. Top 31 compounds were additionally examined for pan-assay interference substructures using PAINS-Remover server [59]. All compounds passed the filter successfully.

## Data Availability

Not applicable.

## Supplementary Materials

The following supporting information can be downloaded at: https://www.mdpi.com/article/10.3390/ijms23062908/s1.
