# Supplementary material for: Complex of HIV-1 Integrase with Cellular Ku Protein: Interaction Interface and Search for Inhibitors
Source: Int J Mol Sci. 2022 Mar 8;23(6):2908. doi: 10.3390/ijms23062908 (PMC8951179; doi:10.3390/ijms23062908)
Supplement: Supplementary file 1 [file ijms-23-02908-s001.zip › captions.pdf]

File S1: MASCOT search results for trypsin digested His6-Ku70; **File S2**: MASCOT search results for GluC digested His6-Ku70; File S3: 3D model of the Ku70/Ku80 heterodimer and integrase dimer complex; **Table S1**: List and 2D structures of compounds used for in vitro testing; **Table S2**: Oligonucleotides used for preparation of prokaryotic and eukaryiotic protein expression vectors; **Figure S1**: Quantification analysis of WB presented at Figure 2C. ** = adjusted *p*-value < 0.01; **** = adjusted *p*-value < 0.0001. **Figure S2**: Ku docking pocket and Y021-2376 docking results.
