# Supplementary material for: Complex of HIV-1 Integrase with Cellular Ku Protein: Interaction Interface and Search for Inhibitors
Source: Int J Mol Sci. 2022 Mar 8;23(6):2908. doi: 10.3390/ijms23062908 (PMC8951179; doi:10.3390/ijms23062908)
Supplement: Supplementary file 1 [file ijms-23-02908-s001.zip › Table S1.pdf]

Table S1. List and 2D structures of compounds used for in vitro testing. ChemDiv ID, SMILES, Molecular Weight, and 2D structure of each compound are indicated.

ChemDiv ID	SMILES	Molecular Weight	2D-Structure
**Y021-2376**	CC1=CC2=C3N(C(=O)C2=O)C(C)(C)C=C(CN2CCN(CC2)C(=O)C2COC4=C(O2)C=CC=C4)C3=C1	487.56	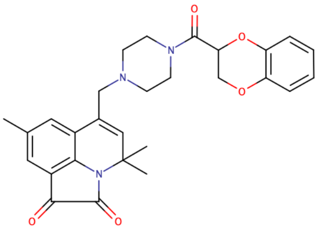
**G902-0998**	CC(CCC1=CC=CC=C1)NC(=O)C1CCN(CC1)C1=NC=NC2=C1C(=NO2)C1=CC=CC(F)=C1	473.55	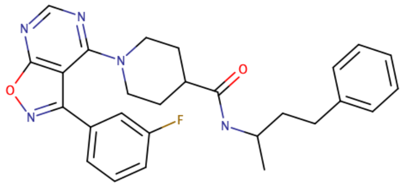
**M337-0620**	CCN1CCN(CC2=CC=C(NC(=O)CN3C=CC4=CC=C(C=C34)C3=NN=C(CC)O3)C=C2)CC1	472.6	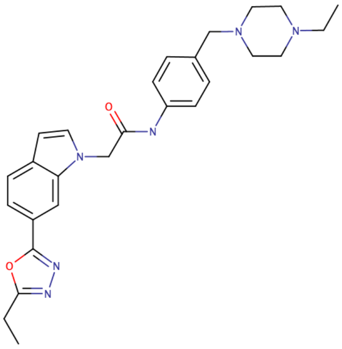
**E717-0291**	CCCN1C2=CC=CC=C2C2=C1C(=O)N(CC(=O)NC1=CC=C(CN3CCN(CC)CC3)C=C1)N=C2	486.62	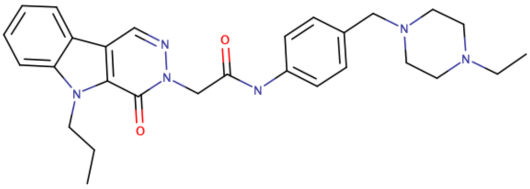
**S642-0153**	CN(CCNC(=O)CN1C(C2=CC=CC=C2)C2(CCCCC2)C1=O)C1CCCCC1	411.59	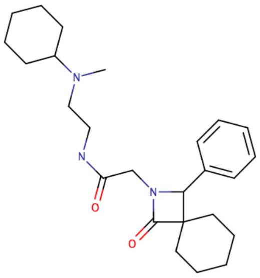
**8019-0500**	O=C(NC1=CC=C(C=C1)C(=O)N1CCOCC1)C(=O)C1=C(NC2=CC=CC=C12)C1=CC=CC=C1	453.5	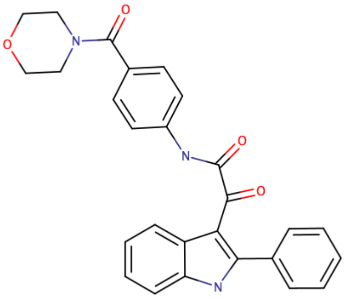

**Y020-4072**	CCN1CCN(CC1)C1=NC=C(C(C)=N1)C1=CC(=O)NC(=N1)N1CCC(CC2=CC=CC=C2)CC1	473.63	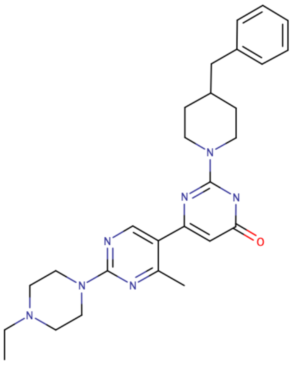
**Y040-3113**	CC1=C(C)C2=CC=C(OCC(=O)N3CCN(CC4=CC5=C(OCO5)C=C4)CC3)C(C)=C2OC1=O	464.52	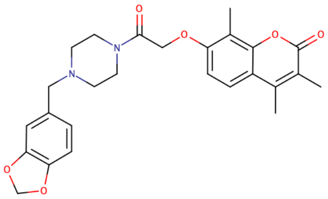
**3272-0701**	O=C(N\N=C\C1=CC=C2CCC3=C2C1=CC=C3)C1=CC=C(CN2CCOCC2)C=C1	399.5	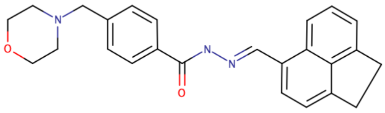
**F828-3136**	CCN1CCN(CC1)C(=O)C1CCN(CC1)S(=O)(=O)C1=C(C)N(N=C1C)C1=CC=C(C)C=C1	473.64	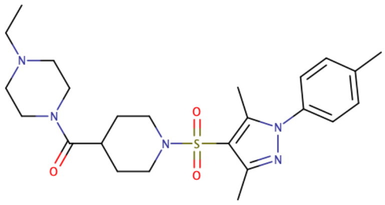
**D715-2836**	COC1=CC=CC2=C1C=C(N2)C(=O)NCCCC(=O)N1CCN(CC2=CC=CC=C2)CC1	434.54	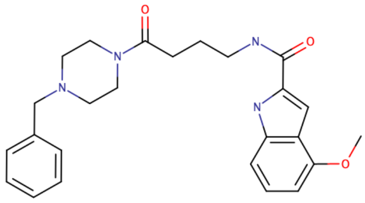
**P435-1819**	COC1=CC=C(C=C1)C1=CC(C(=O)N2CCC(CC2)C2=NC(C(=O)N3CCCC3)=C(C)O2)=C(C)N1C	490.61	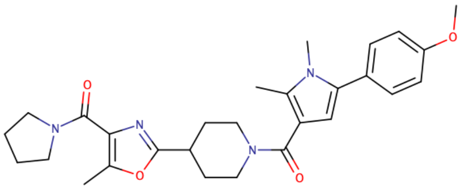
**T622-0507**	CC(C)CN1CCN(CC1)C1CCOC2(CCN(CC2)S(=O)(=O)CC2=CC=CC=C2)C1	449.66	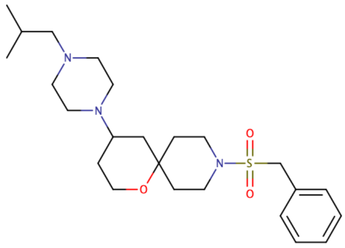
**S722-1010**	COC1=CC=C2C=C(NC2=C1)C(=O)N1CCC(CC2=CC=C(CN3CCN(C)CC3)C=C2)CC1	460.62	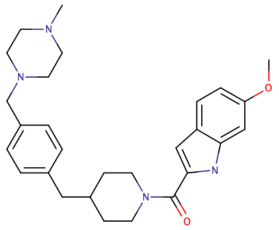

**C880-1498**	CC1=CC=C(C)C(CN2C3=CC=CC=C3C3=C2C(=O)N(CC(=O)NCCN2CCOCC2)N=C3)=C1	473.58	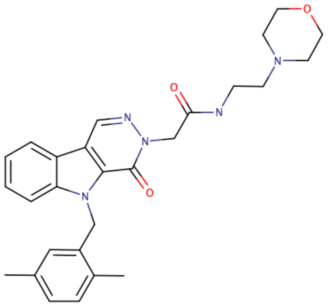
**D481-2269**	O=C(CN1CCN(CC1)C1=CC=CC=C1)NC1=NC(=CC(=N1)C1=CC=CC=C1)C1=CC=CC=C1	449.56	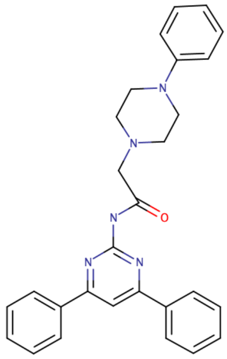
**Y041-8183**	COC1=C(OC)C=C(CN2CCC(CC2)NC(=O)CNC2=NC(C)=CC(C)=N2)C=C1	413.52	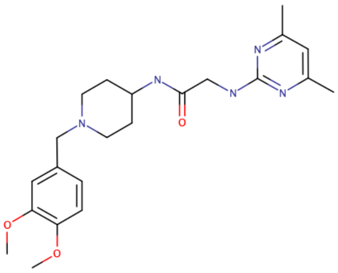
**F681-0474**	CN1C(=O)C(=NC2=CC=CC=C12)N1CCCC(C1)C(=O)NCC1CCN(CC2=CC=C(C)C=C2)CC1	487.65	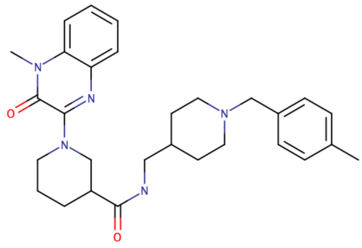
**F382-1633**	CC1=NOC(=C1C)C1=CC(=C(C)C=C1)S(=O)(=O)CCC(=O)N1CCN(CC1)C1CCCCC1	473.638	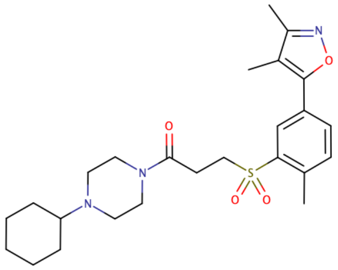
**G614-0077**	CC1=CC(OCC(=O)NCCCN2CCOCC2)=NC2=C1C(=NN2C1=CC=CC=C1)C1=CC=CC=C1	485.59	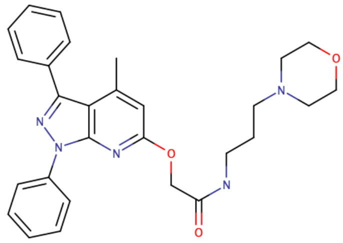
**L332-0248**	COC1=CC=C(C)C=C1NC(=O)NCCC1CCN(CC1)C(=O)NC1CCCCC1	416.57	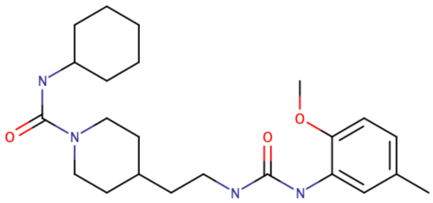

**C618-0810**	CC1=CC=C2OC(=O)C3=C(N(CC(=O)NCCN4CCN(CC5=CC=CC=C5)CC4)N=C3)C2=C1	459.55	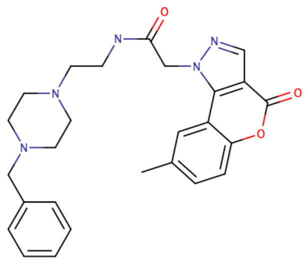
**D718-1195**	CN1CCN(CC(=O)N2CCNC(=O)[C@@H]2CC(=O)NC2=CC=CC3=CC=CC=C23)CC1	423.52	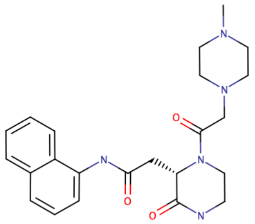
**C387-2091**	COC1=C(NC2=NC3=CC=CC=C3C(=C2)C(=O)NCCCN2CCCC(C)C2)C=CC=C1	432.57	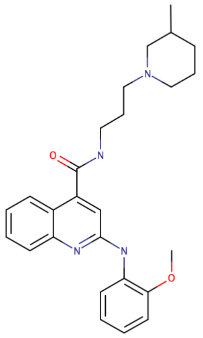
**C782-0380**	CCOC1=CC=C2N=C(SC2=C1)N1CCCC(C1)C(=O)NC1CCN(CC2=CC=CC=C2)CC1	478.66	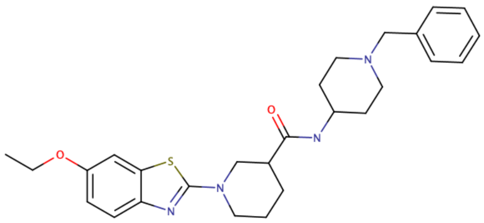
**G415-4813**	COC1=CC=CC=C1CNC1=NC(C)=C(C=N1)C(=O)NC1CCN(CC2=CC=CC=C2)CC1	445.57	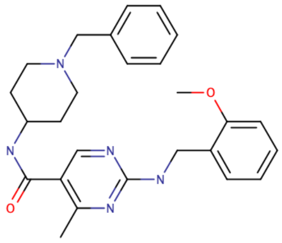
**S497-1413**	CC1=NN(CCC(=O)N2CC(C2)OC2=C(C=CC=C2)C2=NC(=NO2)C2=CC=CC=C2)C(C)=C1	443.51	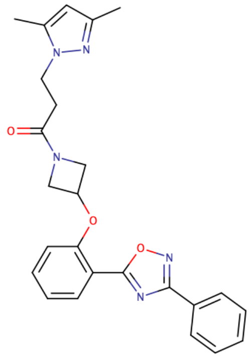

**C525-0837**	COC1=CC(\C=C\C(=O)NCCN2CCC(CC2)N(C)C2CCCCC2C)=C(OC)C=C1	533.67	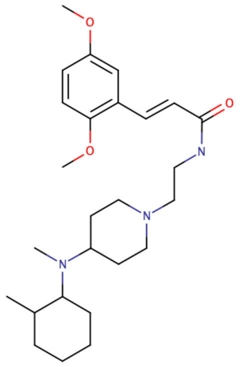
**G267-0963**	CC1=NN(C(C)=C1CCC(=O)NC1=CC=CC(CN2CCCC2)=C1)C1=CC=C(N=N1)N1CCCCC1	487.65	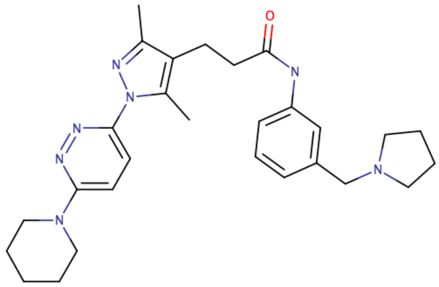
**F383-0897**	COC1=C(C=C(CN2C(=O)C3CCCCC3C2=O)C=C1)S(=O)(=O)N1CCC(=CC1)C1=CC=CC=C1	494.61	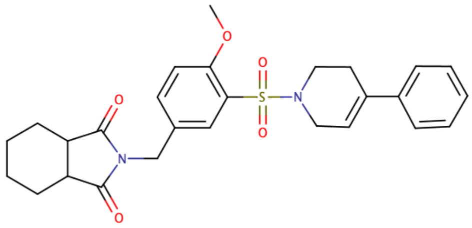
**Y043-6815**	C[C@H](NC(=O)N1CCN(CC2=CC=CC=C2)CC1)C(=O)NC1=CC=C2NC=CC2=C1	405.5	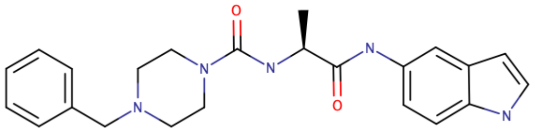
