# Supplementary material for: Complex of HIV-1 Integrase with Cellular Ku Protein: Interaction Interface and Search for Inhibitors
Source: Int J Mol Sci. 2022 Mar 8;23(6):2908. doi: 10.3390/ijms23062908 (PMC8951179; doi:10.3390/ijms23062908)
Supplement: Supplementary file 1 [file ijms-23-02908-s001.zip › Table S2_new.pdf]

Table S2. Oligonucleotides used for preparation of prokaryotic and eukaryiotic protein expression vectors.

Name	Oligonucleotide sequence (5'-3')
Ku70-78_STOP	GTGTACATCAGTAAGATCATAAGCAGTTAGCGAGATCTCTTGGC
Ku70-78_STOP_anti	GCCAAGAGATCTCGCTAACTGCTTATGATCTTACTGATGTACAC
Ku70-64_STOP	GTTGACACCTTTTGACATGAGCTAGCAGTGTATCCAAAGTGTGTACA
Ku70-64_STOP_anti	TGTACACACTTTGGATACACTGCTAGCTCATGTCAAAAGGTGTCAAC
Ku70-44_STOP	GTTTGATTTTTTTGGTTGATGCCTAGAAGGCTATGTTTGAATCTCAGAGT
Ku70-44_STOP_anti	ACTCTGAGATTCAAACATAGCCTTCTAGGCATCAACCAAAAAAATCAAAC
Ku70_Δ51-57/insAG	GGTTGATGCCTCCAAGGCTATGTTTGAAGCTGGAACACCTTTTGACATG
Ku70_Δ51-57/insAG _anti	CATGTCAAAAGGTGTTCCAGCTTCAAACATAGCCTTGGAGGCATCAACC
Ku70_Q65A/Q68A	GATCTTACTGATGTACACACTTGCGATACACGCGATGCTCATGTCAAAAGGTGTC
Ku70_Q65A/Q68A_anti	GACACCTTTTGACATGAGCATCGCGTGTATCGCAAGTGTGTACATCAGTAAGATC
Ku70_S69A/I72A	GATCACTGCTTATGATCTTACTGGCGTACACAGCTTGGATACACTGGATGCTCATGTC
Ku70_S69A/I72A_anti	GACATGAGCATCCAGTGTATCCAAGCTGTGTACGCCAGTAAGATCATAAGCAGTGATC
Ku70_S73A/I76A	CCAAGAGATCTCGATCACTGCTTGCGATCTTAGCGATGTACACACTTTGGATACACT
Ku70_S73A/I76A_anti	AGTGTATCCAAAGTGTGTACATCGCTAAGATCGCAAGCAGTGATCGAGATCTCTTGG
Ku70_S69A	TGATCTTACTGATGTACACAGCTTGGATACACTGGATGCTCATG
Ku70_S69A_anti	CATGAGCATCCAGTGTATCCAAGCTGTGTACATCAGTAAGATCA
Ku70_I72A	ATCACTGCTTATGATCTTACTGGCGTACACACTTTGGATACACTGG
Ku70_I72A_anti	CCAGTGTATCCAAAGTGTGTACGCCAGTAAGATCATAAGCAGTGAT
Ku70_S73A	CTCGATCACTGCTTATGATCTTAGCGATGTACACACTTTGGATACAC
Ku70_S73A_anti	GTGTATCCAAAGTGTGTACATCGCTAAGATCATAAGCAGTGATCGAG
Ku70_I76A	CAAGAGATCTCGATCACTGCTTGCGATCTTACTGATGTACACACTT
Ku70_I76A_anti	AAGTGTGTACATCAGTAAGATCGCAAGCAGTGATCGAGATCTCTTG
siCtr_s	AGGUCGAACUACGGGUCAAdTsdT
siCtr_as	UUGACCCGUAGUUCGACCUdTsdT
siKu70_s	GUGCAAAACGAAUUCUAGAdTsdT
siKu70_as	UCUAGAAUUCGUUUUGCACdTsdT
