# Supplementary figures and images for: Complex of HIV-1 Integrase with Cellular Ku Protein: Interaction Interface and Search for Inhibitors
Source: Int J Mol Sci. 2022 Mar 8;23(6):2908. doi: 10.3390/ijms23062908 (PMC8951179; doi:10.3390/ijms23062908)

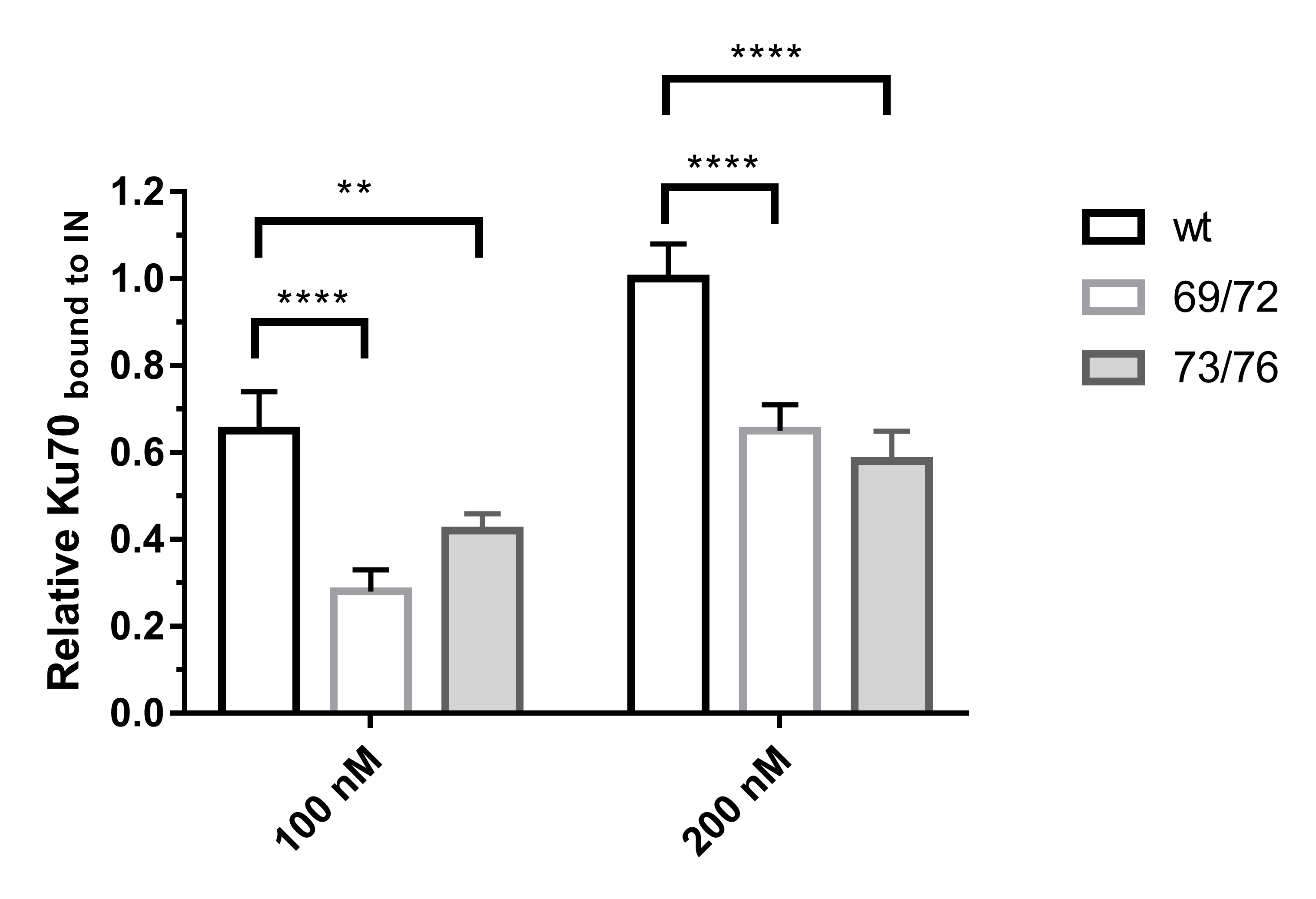

Supplement: Supplementary file 1 [file ijms-23-02908-s001.zip › fig_S1.tif]

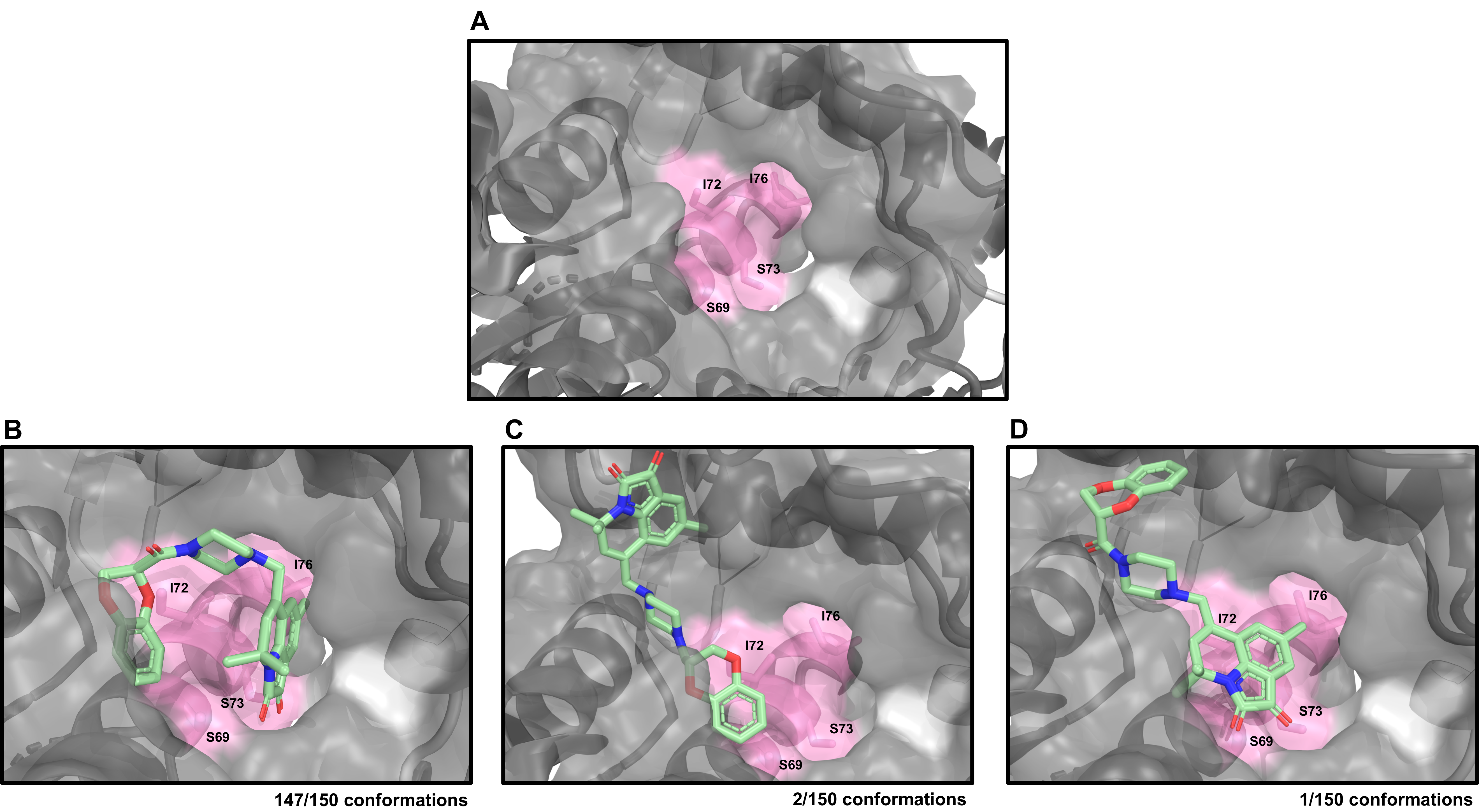

Supplement: Supplementary file 1 [file ijms-23-02908-s001.zip › Fig_s2.png]
